# Acute Effects of Low vs. High Inertia During Flywheel Deadlifts with Equal Force Impulse on Vertical Jump Performance

**DOI:** 10.3390/s25041125

**Published:** 2025-02-13

**Authors:** Athanasios Tsoukos, Margarita Tsoukala, Dimitra Mirto Papadimitriou, Gerasimos Terzis, Gregory C. Bogdanis

**Affiliations:** School of P.E. and Sport Science, National and Kapodistrian University of Athens, 172 37 Athens, Greece; tsoukalamarg8@gmail.com (M.T.); mirto.papadim@gmail.com (D.M.P.); gterzis@phed.uoa.gr (G.T.)

**Keywords:** kinetics, force platform, countermovement jump, drop jump, muscle power

## Abstract

Background: Flywheel resistance training has gained popularity due to its ability to induce eccentric overload and improve strength and power. This study examined the acute effects of low- (0.025 kg·m^2^) versus high-inertia (0.10 kg·m^2^) flywheel deadlifts, matched for force impulse, on the countermovement jump (CMJ) performance, reactive strength index (RSI) during drop jumps (DJs), and rating of perceived exertion (RPE). Methods: Sixteen trained participants (twelve men, and four women) performed three conditions in a randomized, counterbalanced order: low-inertia (LOW), high-inertia (HIGH), and control (CTRL). In the LOW and HIGH conditions, we used force plates to measure and equalize the force impulse in the two conditions (HIGH: 20182 ± 2275 N∙s vs. LOW: 20076 ± 2526 N∙s; *p* > 0.05), by calculating the number of deadlift repetitions required to achieve it (HIGH: 5 repetitions and LOW: 9.8 ± 0.4 repetitions). The RSI and CMJ performance were measured pre-exercise, immediately post-exercise, and at 3, 6, 9, and 12 min post-exercise. Results: Both the RSI and CMJ performance improved equally after LOW and HIGH flywheel deadlifts compared to baseline and CTRL (*p* < 0.01). Specifically, the RSI increased from baseline at 3 to 12 min in both conditions (LOW: 12.8 ± 14.9% to 15.4 ± 14.8%, HIGH: 12.1 ± 17.0% to 12.2 ± 11.7%, *p* < 0.01), while the CMJ increased from 3 to 9 min in LOW (4.3 ± 3.2% to 4.6 ± 4.7%, *p* < 0.01) and from 6 to 9 min in HIGH (3.8 ± 4.2% to 4.2 ± 4.9%, *p* < 0.05). No significant differences were observed between LOW and HIGH conditions (*p* > 0.05), suggesting similar effectiveness of both inertial loads for enhancing performance. The RPE increased similarly after both conditions from baseline to immediately post-conditioning (LOW: from 2.2 ± 1.2 to 5.8 ± 1.4, HIGH: from 1.5 ± 1.0 to 6.1 ± 1.5, *p* < 0.01) and decreased by the end of the session, although values remained higher than baseline (LOW: 4.1 ± 1.4, *p* < 0.01, HIGH: 4.5 ± 2.0, *p* < 0.01). Conclusions: These findings highlight the potential of flywheel deadlift exercise as an effective method to potentiate explosive performance of the lower limbs, regardless of inertia, provided that the total force impulse is equal.

## 1. Introduction

Flywheel resistance training is an innovative method offering several advantages over the traditional approach. These advantages include portability, improved safety, consistent resistance across all planes and axes, and the absence of sticking regions and breaking phases in the concentric part of motion [1,2,3]. Additionally, resistance automatically adjusts to the participants’ fatigue level and can generate an eccentric overload [1,2]. Eccentric overload occurs when the eccentric force or power exceeds the concentric force or power [1]. When a participant is using a flywheel device, resistance is generated by the flywheels’ moment of inertia through its rotation, rather than by gravity, making it an effective method of exercise even in zero-gravity environments [4]. During the concentric phase, such as when rising from a squat position by extending the knees, the flywheel rotation stores kinetic energy within the system [5]. In the subsequent eccentric phase, as the participant lowers by flexing the knees, the previously generated kinetic energy is absorbed by the lower limb muscles [5]. At this phase, the eccentric muscle action acts like a brake, decelerating the downward motion [6]. However, it is important to note that the use of flywheel devices does not inherently lead to eccentric overload. It has been mentioned that the magnitude of eccentric load that is applied during flywheel training is largely dependent on the concentric velocity generated by the participant and the braking strategy employed [1,7,8,9]. Therefore, to generate eccentric overload, the participant should decelerate or attempt to suddenly brake the downward movement in the last third of the eccentric phase [1,7,8,9].

A flywheel exercise can be utilized as a conditioning activity during complex training to provide a stimulus that enhances the subsequent power performance [10], a phenomenon known as post-activation performance enhancement (PAPE) [11,12,13,14], which underpins the principles of complex training [15,16,17]. However, the conditioning activity can also induce fatigue, limiting these improvements [18], and the interaction between PAPE and fatigue ultimately determines the subsequent muscle performance [18]. Research has shown that this interaction is influenced by factors such as the subjects’ characteristics (e.g., muscular strength [19,20], training background [21,22], and muscle fiber type distribution and/or area [23]), the specific characteristics of the conditioning activity (e.g., volume, intensity, muscle length, type of muscle contraction, tempo, and movement velocity loss) [12,18,24,25,26,27], recovery time between the conditioning activity and the subsequent performance [25,28], and the biomechanical similarity between the conditioning activity and the following exercise [29].

In flywheel resistance training, the moment of inertia refers to the resistance provided by the rotating flywheel and dictates the relative intensity of exercise [1,2,6]. Unlike traditional resistance training, where the resistance is determined by the mass of the barbell and plates, flywheel inertia is influenced by the mass, dimensions, and mass distribution of the rotating disk [1,6]. Higher moments of inertia (>0.10 kg ∙ m^2^) require greater force generation, whereas lower moments of inertia (0.025 kg ∙ m^2^) allow higher velocities and may elicit different neuromuscular responses [2,30]. Additionally, studies have demonstrated that different moments of inertia in flywheel resistance squat exercise (ranging from 0.025 to 0.25 kg∙m^2^) lead to varying levels of force, torque, velocity, power, and coordination [30,31,32,33,34,35], thereby inducing different amounts of fatigue [34]. Generally, when using different moments of inertia in the flywheel squat or half-squat exercise (e.g., 0.03–0.12 kg·m^2^) as a conditioning activity, the optimum recovery time is between 3 and 9 min, and two to four sets of approximately 6–7 repetitions are necessary for power performance enhancement [10,36,37,38]. When comparing different moments of inertia of the flywheel on the PAPE effect research has shown inconsistent results [36,37,38]. Beato et al. [36] used moderate- and high-inertia flywheels (0.03 kg·m^2^ vs. 0.06 kg·m^2^) in the squat exercise and found equal improvements on long jump, CMJ, and change of direction performance [36]. In contrast, Fu et al. [37] investigated the PAPE effect of squat flywheel exercise on CMJ performance under three different inertial loads (0.041 kg·m^2^, 0.057 kg·m^2^; and 0.122 kg·m^2^) and found that the PAPE of the vertical explosive force increased with increasing inertial load, but the PAPE effect on the horizontal explosive force was not evident at the highest inertial load. In agreement with that, Shi et al. [39] concluded that moderate and heavier moments of inertia (0.0784 and 0.1568 kg·m^2^) in the flywheel squat exercise induce a greater PAPE in CMJs compared to a lower moment of inertia (0.0465 kg·m^2^). Even more, McErlain-Naylor et al. (2021) compared two moments of inertia (0.029 kg·m^2^ and 0.061 kg·m^2^) and found that the higher-inertia flywheel squats were able to acutely enhance the CMJ peak vertical force whereas lower-inertia flywheel squats acutely the enhanced CMJ peak vertical power [38].

However, all the above studies used a fixed number of sets and repetitions (e.g., two to three sets and six repetitions) to compare the different flywheel inertial loads, ignoring the fact that the total work done is increased with the moment of inertia, possibly inducing higher fatigue [34]. We have previously used the force impulse to equate the total work done when comparing the PAPE effect after different types of muscle actions during resistance exercise [25]. This approach isolates the effects of the total work done on the PAPE, and, thus, enables us to study the pure effects of the independent variable, which, in that case, was the muscle action type [25]. In that study, force impulse was defined as the product of the force applied and the time over which this force acts [25]. This methodology has also been successfully applied to equating throwing protocols [40,41]. Thus, this approach may be useful to utilize during the flywheel exercise, in an attempt to isolate the effects of different moments of inertia on the PAPE. In the present study, in contrast with most previous studies which used the squat or half-squat exercise, we used the deadlift exercise on the flywheel device. The deadlift was chosen because it is a hip-dominant exercise that places greater emphasis on the posterior chain compared to the squat. Given the importance of the posterior chain strength and power in athletic performance, we aimed to investigate whether flywheel deadlifts could induce similar or even superior PAPE effects compared to squats. Additionally, we required the participants to perform plantar flexion of the ankles at the end of the deadlift, achieving an “on-toes” final position, making this exercise more specific to the subsequent jump tests. The only previous study which used the deadlift on a flywheel setting [42] showed improvements only in isokinetic hamstrings eccentric torque, but the CMJ or DJ performance was not examined. Thus, the purpose of the present study was to investigate the influence of low (0.025 kg·m^2^) vs. high inertia (0.10 kg·m^2^) during flywheel deadlifts with an equal-force impulse on the CMJ and DJ performance and rating of perceived exertion (RPE). Specifically, we assessed the CMJ performance and reactive strength index (RSI) during a DJ from a box of 15 cm before, immediately after, and at 3, 6, 9, and 12 min following the flywheel exercise to track the time course of changes in lower limb explosive performance.

## 2. Materials and Methods

### 2.1. Participants

Sixteen (twelve men and four women) strength- and power-trained individuals with a minimum of 3 years of experience took part in the study. They all engaged in various sports (recreational gym-based activities; individual sports like track and field, weightlifting, and wrestling; and team sports such as soccer and basketball). Participants’ characteristics were as follows: MEN: age: 21.8 ± 1.2 years, height: 1.78 ± 0.08 m, body mass: 82.4 ± 15.3 kg, % body fat: 13.6 ± 4.6%; WOMEN: age: 24.6 ± 3.2 years, height: 1.68 ± 0.08 m, body mass: 64.3 ± 8.3 kg, % body fat: 25.1 ± 4.3%. Although participants were physically active, they had no prior experience with flywheel devices. Therefore, they performed three familiarization sessions to ensure proper technique during the flywheel deadlift exercise and jump tests before the experimental conditions. Inclusion criteria required that participants (a) abstained from nutritional supplements or drugs, (b) had no musculoskeletal injuries within the past year, (c) were non-smokers, (d) refrained from training at least 48 h prior to each lab session, and (e) maintained consistent dietary intake 24 h before each test. After receiving a detailed explanation of the protocol, risks, and their right to withdraw at any time, participants provided written informed consent. The study was approved by the Institutional Review Board (Approval no. 1646/12-06-2024) with all procedures following ethical guidelines World Medical Association Helsinki declaration (1964, revised 2013).

### 2.2. Research Design

**Figure 1:**
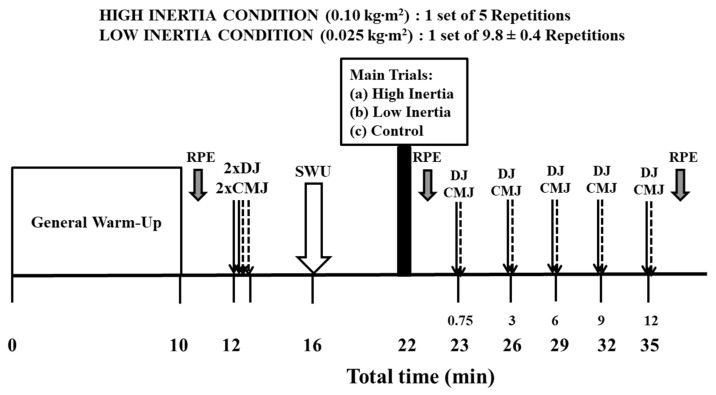
Schematic representation of the experimental protocol. DJ: drop jump from a 15 cm box. CMJ: countermovement jump. SWU: Specific warm-up using five submaximal deadlifts on the flywheel device. RPE: rating of perceived exertion.

A repeated-measures design was used to investigate the acute effects of low (0.025 kg·m^2^) versus high flywheel inertia (0.10 kg·m^2^) during deadlifts performed on a flywheel device (kBox 5, Exxentric, AB TM, Bromma, Sweden) with equated-force impulse on CMJ performance and reactive strength index (RSI) measured during drop jump (DJ). The study involved three preliminary sessions, two experimental conditions and one control (CTRL) condition, organized in a randomized and counterbalanced order. A Latin square design was used to randomize the order of conditions across participants. In the first preliminary session, participants underwent anthropometric assessments and were familiarized with the flywheel deadlift exercise, and the DJ and CMJ exercises. The second preliminary session focused on familiarizing participants with maximum deadlifts against low and high flywheel inertia, and with both jump types. During the third preliminary session, participants performed five deadlifts with either low or high inertia in random order with full recovery. This session aimed to equate protocols based on force impulse (area under the force curve) of the vertical ground reaction force [25]. During the experimental conditions (Figure 1), participants completed a warm-up followed by two CMJs and two DJs from a 15 cm box with 15 s rest intervals. They then performed flywheel deadlifts with either low or high inertia, with the number of repetitions being individualized based on force impulse equalization. CMJ and RSI performance in DJ was reassessed immediately after the deadlifts, and again at 3, 6, 9, and 12 min post-exercise. In the control condition, participants did not perform deadlifts; instead, they rested on the flywheel device and only performed the CMJs and DJs.

### 2.3. Flywheel Deadlifts

The flywheel deadlift was performed on a flywheel device (kBox 5, Exxentric, AB TM, Bromma, Sweden). Participants stood barefoot on two force platforms positioned on the flywheel box, with their feet hip-width apart. The exercise began from the bottom position, with the lower back straight (not arched) and knees bent. Participants grasped the specific barbell of the device, which was positioned around mid-shin height. They then pulled the barbell upwards as forcefully as possible, driving it toward the body until reaching an upright position. Participants were also instructed to plantar flex the ankle joint, achieving a “on-toes” final position. This was carried out to engage the gastrocnemius and soleus muscles.

### 2.4. Equalization of Force Impulse in the HIGH and LOW Conditions

During the third preliminary session, participants performed five deadlifts with either LOW or HIGH inertia in a randomized order. The purpose of this session was to equalize the protocols by measuring the force impulse (area under the force curve) of the vertical ground reaction force [20]. After the standardized warm-up, participants stood in the appropriate stance (as described above) on two force plates (one for each foot) positioned on the flywheel box, and performed five submaximal repetitions against either LOW (0.025 kg·m^2^) or HIGH (0.10 kg·m^2^) inertia. This served as a specific warm-up. Six minutes after the end of the specific warm-up, participants performed five maximal repetitions with each inertia condition in random and counterbalanced order, with a 15-minute rest interval between sets. The total force impulse of the vertical ground reaction force (including body weight) was measured for each inertia condition and used to equalize protocols. The total force impulse during the five repetitions with HIGH inertia was used to standardize the conditioning exercise for the LOW inertia condition. Table 1 shows the average number of repetitions performed in the HIGH- and LOW-inertia main conditions to ensure that the total impulse was similar across both. We used five repetitions in the HIGH-inertia condition as reference for equalizing the number of repetitions in the LOW inertia, as a pilot study indicated that five repetitions generate a force impulse of approximately 20 kN∙s, which has been shown to induce post-activation performance enhancement effect [25]. Ground reaction force was recorded using two dual-axis force plates (PS-2142; PASCO Scientific, Roseville, CA, USA). These force plates were connected and synchronized to a PC via an interface (SPARKlink Air; PS-2011; PASCO Scientific, Roseville, CA, USA), with a sampling frequency of 1000 Hz. The data were then filtered and processed using a customized recording template in PASCO Capstone software (v. 2.6; PASCO Scientific) [43]. In addition to force impulse, we calculated the total time under tension (TUT) and the mean force (MF) applied during deadlifts performed with HIGH and LOW inertia.

### 2.5. General and Specific Warm-Up

Participants completed a standardized warm-up protocol before each preliminary or experimental session. This began with 5 min of light cycling on a cycle ergometer at a resistance of 50–60 watts, followed by 5 min of dynamic stretching and exercises including walking quad stretches, knee lifts to chest, core-focused lunges with a swing, bodyweight squats, and one-leg deadlifts, all targeting the legs and lower back muscles. Moreover, five repetitions of good mornings using a 15 kg barbell, three submaximal bodyweight jump squats, and six pogo jumps were performed. The specific warm-up included 1 set of 5 submaximal repetitions at the target inertia for either HIGH or LOW condition.

### 2.6. Main Trials

The two main experimental trials, along with the control condition (CTRL), were conducted in a randomized, counterbalanced order, with a 5- to 7-day interval between sessions. The conditions were defined as follows:

(a) HIGH moment of inertia condition (HIGH): Participants performed 1 set of 5 repetitions with a moment of inertia of 0.10 kg·m^2^.

(b) LOW moment of inertia condition (LOW): Participants completed one set with the predetermined number of repetitions identified during the third preliminary session, which matched the force impulse of the HIGH condition. As shown in Table 1, participants performed an average of 9.8 ± 0.4 repetitions in the LOW condition.

(c) Control condition (CTRL): Participants did not engage in any conditioning exercise; instead, they completed only the vertical jump tests (CMJ and DJ).

Each experimental trial began with a standardized general warm-up. Following this, participants performed two DJs and two CMJs with the average of these jumps recorded as the baseline measure. After a 4 min rest, participants completed the specific warm-up. This was followed by a 6-minute rest. Subsequently, participants either performed the conditioning exercise (HIGH or LOW) with maximal effort or rested in a seated position for 5 min (CTRL). All repetitions were counted from the flywheel at a standstill. Participants initiated each trial from the bottom position with the barbell at mid-shin height, the lower back straight (not arched), and knees bent (as described above), without performing additionally preparatory repetitions to accelerate the inertia. Each repetition was executed with maximal effort from the first repetition. Furthermore, considering the potential effects of fatigue on post-activation performance enhancement (PAPE), we intentionally avoided additional repetitions before the main set to prevent unnecessary fatigue that could have influenced acute performance outcomes. Following the conditioning exercise, the DJ and CMJ were re-assessed at 45 s, and again at 3, 6, 9, and 12 min post-conditioning.

### 2.7. Countermovement Jump and Drop Jump Performance

CMJ performance was assessed using an optical measurement system with a sampling frequency of 1,000 Hz (Optojump Next; Microgate, Bolzano, Italy) that measured flight time. During the CMJ, athletes were asked to jump as high as possible with their arms on the hips, while maintaining the same body position during take-off and landing [44,45]. The ICC for CMJ was 0.993 (95%CI: 0.983–0.997; *p* < 0.001).

For the drop jump, participants stood on a 15 cm box with their feet hip-width apart near the edge. They stepped off gently, allowing gravity to bring them down without actively jumping. Upon landing on the balls of the feet, they immediately rebounded into a maximal vertical jump, aiming to minimize ground contact time [44,46]. Their arms were maintained on their hips throughout the movement. RSI was calculated using the formula: RSI = $\frac{jump\;height\;(m)}{Contact\;time\;(s)}$. The ICC for DJ was 0.982 (95%CI: 0.959–0.993; *p* < 0.001).

### 2.8. Rating of Perceived Exertion (RPE)

Rating of perceived exertion (RPE) was evaluated using a Borg scale (CR10-scale) one minute after completing the warm-up, immediately following the flywheel deadlifts and after performing the DJ and CMJ at the 12th minute of recovery. Each participant was individually called by the authors and shown a visual representation of the scale, and the response was recorded [47].

### 2.9. Statistical Analysis

Statistical analyses were performed using the SPSS Statistics Ver. 23 (IBM Corporation, Armonk, NY, USA). Data are presented as means ± standard deviations (SD). A two-way repeated-measures ANOVA was conducted to assess differences across conditions and time points, with a 3 × 6 design (conditions: HIGH, LOW, and CTRL vs. time points: pre, 0.75, 3, 6, 9, and 12 min) for all measured variables. In addition, a two-way repeated-measures ANOVA 3 × 2 design (conditions: HIGH and LOW vs. time points: pre and best) was used to examine differences in the best jump performance post-conditioning across conditions. Furthermore, a two-way ANOVA 3 × 3 design with condition was employed to evaluate differences in RPE measurements across the three conditions and the three time points (pre-, post-exercise, and end of protocol). When a significant main effect or interaction was found, Tukey’s post hoc test was applied. Effect sizes for main effects and interactions were evaluated using partial eta squared (η^2^p), classified as small (0.01 to 0.059), moderate (0.06 to 0.137), and large (>0.137). T-tests were used to examine differences between the conditioning activities of the HIGH or LOW conditions (force impulse, mean force, and time under tension). For pairwise comparisons, effect size was calculated using Hedges’ g, categorized as small (<0.3), medium (0.3–0.8), and large (>0.8). Statistical significance was set at *p* < 0.05.

## 3. Results

### 3.1. Characteristics of the Flywheel Deadlifts

Table 1 summarizes the differences in characteristics between the HIGH- and LOW-inertia deadlifts. T-tests revealed no significant difference in the total force impulse between conditions (*p* = 0.68; Hedges’ g = 0.04), indicating that the protocols were equalized. However, both the number of repetitions (*p* < 0.001; Hedges’ g = 16.0) and TUT (*p* < 0.001; Hedges’ g = 0.8) were significantly higher in the LOW-inertia condition compared to the HIGH-inertia condition, while the mean force was lower (*p* < 0.001; Hedges’ g = 0.5).

### 3.2. Time Course of RSI in the Main Conditions

**Figure 2:**
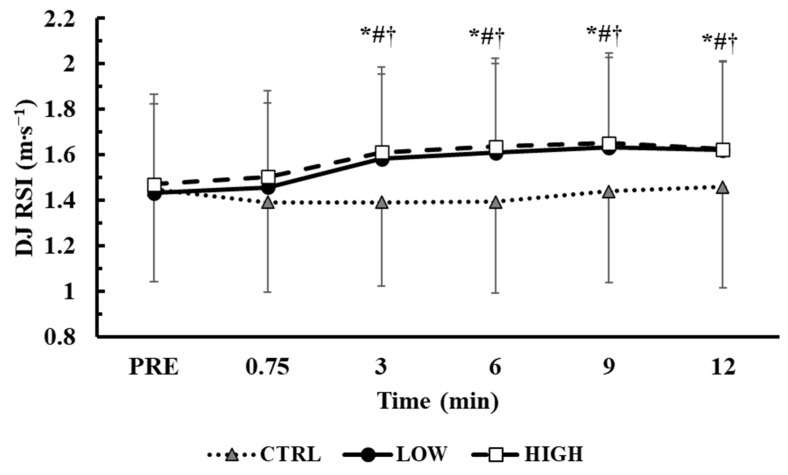
Time course of changes of the RSI index during drop jumps from a 15 cm box. *: *p* < 0.01 from PRE in LOW; #: *p* < 0.01 from PRE in HIGH; †: *p* < 0.01 from CTRL in the corresponding time point in both LOW and HIGH conditions.

The time course of the RSI performance changes during the main trials is presented in Figure 2. The baseline RSI values were similar across conditions (*p* = 1.0). The two-way ANOVA (3 conditions × 6 time points) showed a significant interaction effect between condition and time (*p* < 0.001, η^2^p = 0.24). Tukey’s post hoc tests revealed that the RSI index improved from baseline in both the LOW and HIGH conditions from the 3rd (LOW: +12.8 ± 14.9%, *p* < 0.01, Hedges’ g = 0.38; HIGH: +12.1 ± 17.0%, *p* < 0.01, Hedges’ g = 0.36) to the 12th minute of recovery (LOW: +15.4 ± 14.8%, *p* < 0.01, Hedges’ g = 0.47; HIGH: +12.2 ± 11.7%, *p* < 0.01, Hedges’ g = 0.38).

Compared to the CTRL condition, the RSI values were similarly higher in both LOW and HIGH from the 3rd to the 12th minute of recovery (LOW: *p* < 0.01, Hedges’ g = from 0.38 to 0.53; HIGH: *p* < 0.01, Hedges’ g = from 0.39 to 0.60). When taking into consideration the best RSI value during post time points (3 conditions × 2 time points), a significant interaction was also observed (*p* < 0.01, η^2^p = 0.36). Tukey’s post hoc tests revealed that the RSI index improved similarly from baseline in both the LOW and HIGH conditions (LOW: +20.6 ± 14.4%, *p* < 0.01, Hedges’ g = 0.65; HIGH: +19.2 ± 15.6%, *p* < 0.01, Hedges’ g = 0.61), while the RSI was also higher compared with the CTRL condition (LOW: *p* < 0.01, Hedges’ g = 0.37; HIGH: *p* < 0.01, Hedges’ g = 0.43) during which the RSI was unchanged (Figure 2).

### 3.3. Time Course of CMJ Performance in the Main Conditions

**Figure 3:**
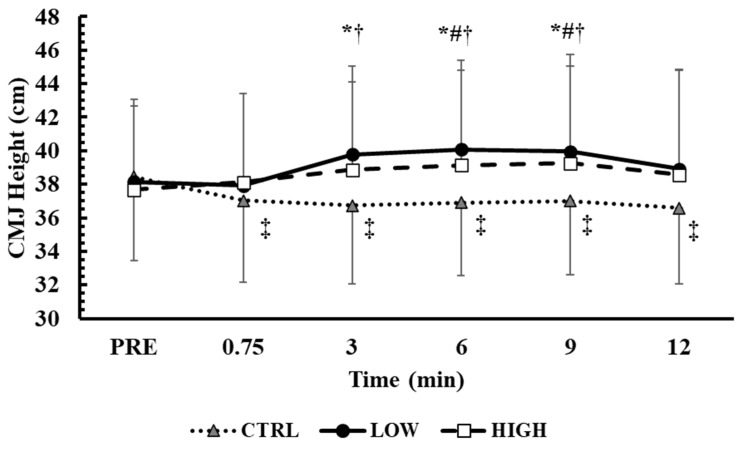
Time course of changes of the CMJ performance. *: *p* < 0.01 from PRE in LOW; #: *p* < 0.05 from PRE in HIGH; ‡: *p* < 0.05 from PRE in CTRL; †: *p* < 0.01 from CTRL in the corresponding time point in both LOW and HIGH conditions.

The time course of CMJ performance changes during the main trials is presented in Figure 3. The baseline CMJ values were similar across conditions (*p* = 1.0). The two-way ANOVA (3 conditions × 6 time points) showed a significant interaction effect between condition and time (*p* < 0.001, η^2^p = 0.34). Tukey’s post hoc tests revealed that the CMJ performance improved from baseline in LOW from the 3rd (+4.3 ± 3.2%, *p* < 0.01, Hedges’ g = 0.32) to the 9th minute of recovery (+4.6 ± 4.7%, *p* < 0.01, Hedges’ g = 0.33). On the other hand, the CMJ performance improved in HIGH from the 6th (+3.8 ± 4.2%, *p* < 0.05, Hedges’ g = 0.27) to the 9th (+4.2 ± 4.9%, *p* < 0.05, Hedges’ g = 0.29) minute of recovery. It is important to note that, in the CTRL condition, the CMJ performance was lower compared to baseline at all time points (immediately post: −3.7% ± 3.6%, *p* < 0.05, Hedges’ g = 0.28, 12th: −4.7%± 4.6% *p* < 0.01, Hedges’ g = 0.38).

Compared to the CTRL condition, the CMJ values were similarly higher in both the LOW and HIGH conditions from the 3rd to the 9th minute of recovery (LOW: *p* < 0.01, Hedges’ g= from 0.43 to 0.60; HIGH: *p* < 0.01, Hedges’ g= from 0.35 to 0.43). When taking into consideration the best CMJ value during post time points (3 conditions × 2 time points), a significant interaction was also observed (*p* < 0.01, η^2^p = 0.56). Tukey’s post hoc tests revealed that the CMJ performance improved similarly compared with baseline in both the LOW and HIGH conditions (LOW: +6.9 ± 3.0%, *p* < 0.01, Hedges’ g = 0.49; HIGH: +6.0 ± 5.5%, *p* < 0.01, Hedges’ g = 0.41), while the CMJ was also higher compared with the CTRL condition (LOW: +7.6 ± 5.7%, *p* < 0.01, Hedges’ g = 0.56; HIGH: +5.3 ± 6.5%, *p* < 0.01, Hedges’ g = 0.38). No significant differences were observed between the LOW and HIGH conditions.

### 3.4. Time Course of RPE in the Main Conditions

**Figure 4:**
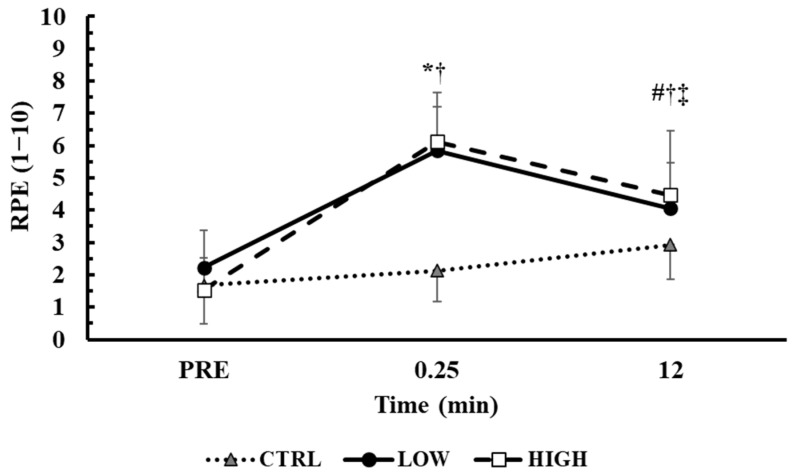
Time course of changes of the RPE. *: *p* < 0.01 from PRE in LOW and HIGH; #: *p* < 0.01 from PRE in all conditions; †: *p* < 0.01 from CTRL in the corresponding time point in both LOW and HIGH conditions; ‡: *p* < 0.01 from 0.25 in LOW and HIGH conditions.

The time course of RPE during the main trials is presented in Figure 4. The baseline RPE values were similar across conditions (*p* > 0.71). The two-way ANOVA (3 conditions × 3 time points) showed a significant interaction effect between condition and time (*p* < 0.001, η^2^p = 0.62). Tukey’s post hoc tests revealed that the RPE was similarly higher from baseline in both LOW and HIGH at immediately post (LOW: from 2.2 ± 1.2 to 5.8 ± 1.4, *p* < 0.01, Hedges’ g = 2.8; HIGH: from 1.5 ± 1.0 to 6.1 ± 1.5, *p* < 0.01, Hedges’ g = 3.5) and at the end of the session (LOW: 4.1 ± 1.4, *p* < 0.01, Hedges’ g = 1.4; HIGH: 4.5 ± 2.0, *p* < 0.01, Hedges’ g = 1.8). Moreover, the RPE decreased at the end compared to immediately post in both the LOW and HIGH conditions (LOW: *p* < 0.01, Hedges’ g = 1.3; HIGH: *p* < 0.01, Hedges’ g = 0.9). At post, both LOW and HIGH had higher values compared to the CTRL condition (*p* < 0.05).

## 4. Discussion

The purpose of this study was to investigate the effects of a low (0.025 kg·m^2^) vs. high moment of inertia (0.10 kg·m^2^) during flywheel deadlifts, matched for force impulse, on the vertical jump performance and rating of perceived exertion (RPE). Both LOW- and HIGH-inertia conditions equally improved the RSI during DJs and the CMJ performance compared to the CTRL condition between 3 to 12 min post-exercise. The RSI increased from baseline at 3 to 12 min in both conditions, while the CMJ performance improved from 3 to 9 min in LOW and from 6 to 12 min in HIGH. No significant differences were found between the LOW and HIGH conditions, indicating a similar effectiveness for inducing PAPE. Furthermore, RPE increased significantly from baseline to immediately after the flywheel exercise in both conditions, decreased by the end of the session, but remained elevated compared to baseline and the CTRL condition.

This study is the first to examine the effects of the flywheel exercise using two distinctly different flywheel moments of inertia, matched for force impulse, on vertical jump performance and RPE. We hypothesized that both inertia conditions would produce a similar PAPE effect, as the training volume, expressed as force impulse, was matched. Our findings confirmed this hypothesis, as the PAPE induced by both protocols was equal and exhibited the same time course in the two conditions. Moreover, the two different flywheel resistances induced similar fatigue, as indicated by the equal RPE values observed throughout recovery. The link between RPE and fatigue has been established during acute resistance exercise [48]. For example, RPE during a repeated-sets protocol of high-velocity parallel squats followed the decline in movement velocity and power output losses [48]. In the present study, the equal RPE values in the HIGH and LOW conditions may indicate similar fatigue levels, which, in this case, seem to depend more on the total training volume than the mean force or number of repetitions.

The findings of the present study align with those of Beato et al. [36] but contrast with other studies [37,39]. In the study of Beato et al. [36], participants performed three sets of six repetitions using low and moderate moments of inertia, with two minutes of recovery between sets. The similar PAPE outcomes observed in their study may be attributed to the similar intensity levels induced by the two moments of inertia which differed only by two-fold (0.03 vs. 0.06 kg·m^2^). Additionally, the inclusion of short recovery periods between sets may have limited the accumulation of a greater force impulse in the moderate-inertia condition compared to the lower-inertia condition. In the present study, two distinctly different loads were used, with the LOW condition having a four-fold lower moment of inertia than the HIGH condition. Moreover, we matched the total work done during the conditioning exercise by equating the force impulse, whereas, in Beato et al. [36] study, total work was obviously different between the two loads. However, the small differences between the two moments of inertia used and the recovery time between sets may have blunted the fatigue in that study, resulting in similar PAPE effects. Therefore, based on the findings of the present study, we propose that the total force impulse is an important variable affecting PAPE, rather than inertia itself. This highlights the importance of considering the total work when designing flywheel training programs.

In contrast, some previous studies [37,39] suggested that higher moments of inertia during flywheel squat exercises induce a higher PAPE effect. Those studies employed multiple sets to induce PAPE. For example, in the study of Fu et al. [37], participants performed four sets of seven repetitions using inertial loads of 0.041 kg·m^2^; 0.057 kg·m^2^, and 0.122 kg·m^2^, whereas, in the study of Shi et al. [39], participants performed three sets of eight repetitions with 3 min rest intervals between sets, using inertial loads 0.0465, 0.0784, and 0.1568 kg·m^2^. It is possible that the lower inertial loads may require a greater number of repetitions or sets to increase the total force impulse sufficiently to induce a PAPE effect comparable to higher inertial loads. Additionally, during flywheel training, producing a specific and concentrated amount of force impulse—especially when using lower inertial loads—may be critical for eliciting a PAPE effect, compared with distributing the force impulse across smaller sets, which might reduce its efficacy. This suggests that there may be a threshold of total force impulse required to effectively induce PAPE.

The finding that the RSI increased from baseline at 3 to 12 min in both conditions, while the CMJ performance improved from 3 to 9 min in LOW and from 6 to 12 min in HIGH, may be influenced by the order of the measurement tests. In the present study, the CMJ performance was assessed following the DJ, which may have induced additional fatigue. These results align with those of Prieske et al. [49] who assessed the PAPE effect on the CMJ performance followed by the DJ performance and found that CMJ height was significantly higher than the CTRL condition, while the RSI during the DJ was not significantly improved.

On the practical applications aspect, the findings of the present study provide guidance for strength and conditioning coaches. Both HIGH- and LOW-inertia flywheel training can be used to elicit PAPE effects, allowing coaches to select the optimum inertia load based on athlete needs or equipment availability. Coaches should focus on matching the force impulse to optimize PAPE, regardless of the inertia load, and use the RPE as a simple and effective tool to monitor fatigue and recovery. These insights can help tailor flywheel training for athletes of different levels and performance goals.

This study has certain limitations which should be considered, First, the sample size and homogeneity of the participants may limit the generalizability of the findings to a broader population. The use of the self-reported RPE is another limitation, as it is subjective and may not fully capture the physiological changes associated with fatigue. Furthermore, while the force impulse was matched across conditions, the mechanical differences between HIGH and LOW inertial loads may not have been fully explored. The order of measurement tests, with the CMJ assessed immediately following the DJ, may have induced additional fatigue prior to the CMJ test, potentially influencing performance. This sequence effect may explain the shorter duration of the CMJ enhancement in comparison to the RSI. Finally, the study used a limited number of exercise modalities, and expanding the variety of exercises or including multiple control conditions could provide a more comprehensive understanding of the effects on performance and fatigue. Future studies should focus on examining the long-term adaptations to flywheel training with HIGH- and LOW-inertia loads, exploring how repeated exposure influences performance and recovery. Moreover, investigating the minimum effective force impulse threshold required to elicit PAPE, as well as optimizing test protocols to minimize fatigue effects on subsequent performance tests, would provide valuable insights. Lastly, incorporating diverse populations, fatigue markers, and varied exercise modalities could enhance generalizability and practical applications.

## 5. Conclusions

This study showed that flywheel exercise using LOW and HIGH inertial loads can elicit a similar PAPE effect when force impulse is matched, highlighting the importance of the total volume rather than of the specific inertia used. Performance improvements were observed between 3 and 12 min of recovery, with LOW inertia eliciting earlier CMJ enhancements (3–9 min) compared to HIGH (6–9 min). These findings suggest that both inertia loads are effective tools for enhancing athletic performance and offer flexibility for strength and conditioning coached to tailor training protocols based on timing and athlete needs. Furthermore, the results underscore the potential of flywheel training as an effective method to induce PAPE, regardless of inertia, provided that the total force impulse is sufficient. This flexibility allows coaches to adapt training strategies based on the equipment available, the athlete’s skill level, and the demands of specific sports or competition schedules. In fitness facilities or rehabilitation clinics where the equipment for equalizing the force impulse is limited, practitioners can approximate force impulse matching by adjusting the number of repetitions based on the moment of inertia of the flywheel used.

## Figures and Tables

**Table 1:** Characteristics of deadlifts performed under HIGH- and LOW-inertia conditions.

	High	Low
**Number of Repetitions**	5 *	9.8 ± 0.4
**Total Impulse (N∙s)**	20,182 ± 2275	20,076 ± 2526
**Time Under Tension (s)**	12.5 ± 1.5 *	13.7 ± 1.7
**Mean Force (N)**	1663 ± 357 *	1491 ± 318

*: *p* < 0.001 from LOW.

## Data Availability

The data are available upon request from the corresponding author.
